# Selenium source and level on performance, selenium retention and biochemical responses of young broiler chicks

**DOI:** 10.1186/s12917-021-02855-4

**Published:** 2021-04-09

**Authors:** Pedro Righetti Arnaut, Gabriel da Silva Viana, Lucimauro da Fonseca, Warley Junior Alves, Jorge Cunha Lima Muniz, James Eugene Pettigrew, Fabyano Fonseca e Silva, Horácio Santiago Rostagno, Melissa Izabel Hannas

**Affiliations:** 1 Department of Animal Science, Federal University of Viçosa, Viçosa, 36570900 Brazil; 2 Production Systems, Natural Resources Institute Finland (Luke), 31600 Jokioinen, Finland; 3 Pettigrew Research Services, Inc., Champaign, IL 61821 USA

**Keywords:** Glutathione peroxidase, Organic trace minerals, Selenium balance, Selenium yeast, Tissue mineralization

## Abstract

**Background:**

Selenium (Se) has been recognized as an essential micronutrient for nearly all forms of life. In recent decades, broiler responses to dietary Se supplemental levels and sources have received considerable attention. On environmental grounds, organic trace mineral utilization in practical broiler feeds has been defended due to its higher bioavailability. In such feeds, trace minerals are provided simultaneously in the same supplement as inorganic salts or organic chelates, a fact commonly ignored in assays conducted to validate organic trace mineral sources. The current assay aimed to investigate growth and biochemical responses, as well as Se retention of growing chicks fed diets supplemented with organic and inorganic Se levels and where the trace minerals (zinc, copper, manganese, and iron) were provided as organic chelates or inorganic salts according to Se source assessed. In so doing, a 2 × 5 factorial arrangement was used to investigate the effects of sodium selenite (SS) and selenium-yeast (SY) supplemented in feeds to provide the levels of 0, 0.08, 0.16, 0.24, and 0.32 mg Se/kg.

**Results:**

Chicks fed selenium-yeast diets had body weight (BW), and average daily gain (ADG) maximized at 0.133 and 0.130 mg Se/kg, respectively. Both Se sources linearly increased (*P* < 0.05) the glutathione peroxidase (GSH-Px) activity in chick blood but higher values were observed in sodium selenite fed chicks (*P* < 0.05). Both Se sources influenced thyroid hormone serum concentrations (*P* < 0.05). Chicks fed SY exhibited greater retention of Se in the feathers (*P* < 0.05). Relative bioavailability of selenium yeast compared with SS for the Se content in carcass, feathers, total and Se retention were, 126, 116, 125 and 125%, respectively. SY supplementation resulted in lower liver Se concentration as Se supplementation increased (*P* < 0.05).

**Conclusions:**

Based on performance traits, the supplemental level of organic Se as SY in organic trace minerals supplement to support the maximal growth of broiler chicks is 0.133 mg Se/kg.

## Background

Selenium (Se) has been recognized as an essential trace element for all forms of animal life. In avian species, Se is required for the synthesis of selenocysteine (Se-Cys), an amino acid present in selenoproteins of biological importance in poultry metabolism such as glutathione peroxidase (GSH-Px), thioredoxin reductase, and iodothyronine deiodinase family [1]. Whereas the first two selenoenzymes modulate antioxidant defenses against reactive oxygen species (ROS) which may damage cell functioning [2–5], the latter family of enzymes participates on thyroid metabolism by converting thyroxin (T4) into triiodothyronine (T3), a hormone involved in the metabolism of proteins, carbohydrates and lipids [6–10]. Even though Se deficiency has been associated with metabolic disorders [11–16], the extent to which chick growth is affected by dietary Se supplementation has been a matter of discussion amongst poultry nutritionists. Previous research findings have demonstrated no effects of Se supplementation on broiler growth performance [3, 17–19], suggesting, therefore, that cereal-based diets could provide the amounts of Se required for physiological needs.

Indeed, cereals utilized in practical feeds contain Se, being 50% found as selenomethionine (Se-Met); however, because Se content of plant cereals are affected by Se content in the soil, the poultry industry supplements Se to avoid nutritional deficiencies [1, 20]. NRC [21] describe Se requirements as 0.15 mg Se/kg which was later suggested by Wang et al. [10]. Conversely, Cai et al. [18] and Cemin et al. [22] recommended the supplemental levels of 0.30 and 0.75 mg Se/kg, respectively. When considering that fast growth genotypes currently raised exhibit higher metabolic rates, and may, therefore, be more susceptible to ROS production, higher amounts of Se are expected to be required to ensure quality chicken meat [18, 23]. Conventionally, Se is supplemented in diets either as inorganic salts such as sodium selenite (SS) or as the organic form of selenium enriched-yeast (SY), which is majorly composed of Se-Met [24]. Because Se present in the selenium yeast is in an organic structure, it is considered less toxic, more digestible, retained and bioavailable than the SS [2, 4, 19, 25, 26].

As well as Se, other trace minerals provided as organic sources are more bioavailable than inorganic sources. When supplied in practical diets, SS is typically used in supplements with inorganic sources of other trace minerals; meanwhile selenium yeast is typically used with organic sources of other trace minerals. In case interactions among minerals may be affected by the form of the dietary minerals, it seems appropriate to assess the optimal dietary concentrations of supplemental selenium for use by the poultry industry in the dietary environment in which each selenium source will typically be used in practice, inorganic selenium with other inorganic trace minerals and organic selenium with other organic trace minerals. We conducted the current study to determine the effect of levels and sources of Se provided by organic and inorganic trace mineral supplements on performance, Se retention and physiological responses of young broiler chicks.

## Results

The analyzed concentrations of Se in SS supplemented diets were 0.123, 0.192. 0.245, 0.356 and 0.428 mg Se/kg, whereas the concentration in the diets supplemented with SY were 0.131, 0.213, 0.271, 0.350, 0.418 mg Se/kg. These values for Se agree with the calculated values for both sources of 0.138, 0.218, 0.298, 0.378 and 0.458 mg of Se/kg.

### Growth performance

No interactions were observed between the micromineral source (inorganic or organic) and Se level (*P* > 0.05) in any of the variables measured in this experiment. There were no main effects of micromineral source on measures of growth performance. Considering data from both sources, the supplemented levels of Se affected (*P* < 0.05) both BW and ADG (Table 1), but the pattern of response was not described by a quadratic regression. The broiler chicks receiving a diet with 0.160 mg Se/kg as SY and organic trace minerals showed higher BW (*P* < 0.05), ADG (*P* < 0.05) and ADFI (*P* < 0.05) compared with the SS and inorganic trace minerals at the same supplementation level. Among the SY treatments, the highest values for BW and ADG were at intermediate dietary Se levels (quadratic: *P* = 0.088 for BW, 0.093 for ADG), (Table 1), and the optimal supplementation levels were estimated at 0.133 and 0.130 mg Se/kg, respectively, through the derivative of the fitted polynomial quadratic models BW_(SY)_ = − 501.8 × ^2^ + 133.2x + 500.5, *r*^2^ = 0.08, and ADG_(SY)_ = − 48.4899 × ^2^ + 12.6437x + 32.0393, *r*^2^ = 0.08. The FC increased linearly (*P* < 0.05) as SY supplementation increased. A lower BW (*P* < 0.05) and ADG (*P* < 0.05) were found for SY supplementation at 0.32 mg Se/kg when compared with 0.16 mg Se/kg, and FC increased linearly (*P* < 0.05) with increasing levels of SY supplementation. As the level of SS supplementation increased, ADG tended to respond quadratically, but the pattern of response differed (*P* < 0.05) from the response to SY level, which reached maximum at 0.16% supplemental Se. The ADFI tended (*P* = 0.090) to decline linearly as dietary level of SS increased. A low incidence of mortality was noticed throughout the trial: one chick fed 0.16 mg Se/kg in the diet supplemented with SS, and other bird fed 0.08 mg Se/kg in the diet supplemented with SY.

**Table 1:** Effects of dietary selenium levels and sources on performance traits of 17-d-old broiler chickens

Selenium source^1^	Selenium levels, mg/kg					Means	SEM^2^	*P-Value*				
	0.0	0.08	0.16	0.24	0.32			Source	Level	S X L^3^	L^4^	Q^5^
***Initial weight, g/bird***												
SS	180.4	180.4	180.5	180.3	180.4	180.4	0.038	0.478	0.999	-	-	-
SY	180.2	180.3	180.1	180.3	180.2	180.2						
***Body weight, g/bird***												
Means	510	490	508	502	494		6.84	0.432	0.019	0.144	0.180	0.977
SS	514	487	497 b	500	498	499					0.378	0.104
SY	506 AB	492 AB	521 Aa	502 AB	490 B	502					0.308	0.088
***Average daily gain, g/bird/day***												
Means	32.9 A	31.0 B	32.9 AB	32.1 AB	31.4 AB		0.672	0.429	0.017	0.132	0.165	0.977
SS	33.3	30.7	31.6 b	31.9	31.7	31.9					0.366	0.097^e^
SY	32.6 AB	31.3 B	34.1 Aa	32.2 AB	30.9 B	32.2					0.283	0.093^f^
***Average daily feed intake, g/bird/day***												
Means	47.3	45.5	47.2	46.4	45.9		0.790	0.661	0.122	0.107	0.847	0.160
SS	48.5 a	45.6	46.1 b	46.4	46.0	46.6					0.090	0.122
SY	46.0 b	45.3	48.2 a	46.4	45.8	46.3					0.847	0.160
***Feed conversion rate, g/g***												
Means	1.43	1.45	1.45	1.45	1.47		0.015	0.238	0.185	0.335	0.018	0.809
SS	1.44	1.46	1.46	1.46	1.45	1.46					0.661	0.449
SY	1.42 B	1.44 AB	1.44 AB	1.44 AB	1.48 A	1.44					0.004	0.616

^1^ *SS* sodium selenite, *SY* selenium enriched-yeast

^2^ Standard error of means

^3^ Interactive effects between selenium and trace minerals sources and selenium levels

^4^ Linear effect of supplementation selenium levels

^5^ Quadratic effect of supplementation selenium levels

^A-B^ Different uppercase letters in the same line and lowercase letters in the same column are different by Tukey test at 5%

^ef^ Different superscripts letter indicates difference in the orthogonal contrasts between equations

### Enzyme activity and hormone concentrations

As shown in Table 2, inorganic trace minerals resulted in higher values of GSH-Px activity in the serum over all Se levels (*P* < 0.01) and at 0.24 mg added Se/kg compared to organic trace minerals. GSH-Px activity in the blood of broiler chicks rose linearly (*P* < 0.05) as supplemented Se level from either source increased (Table 2). There were no treatment effects on superoxide dismutase concentrations. Considering both sources together, Se levels affected free T4 (fT4) and tended to affect the total T3 concentration (*P* = 0.056) and free T3:free T4 (*P* = 0.078), but the patterns of response were not described by quadratic regression. There was a quadratic response (*P* < 0.05) in total T3 concentration with the greatest value where no SS was added. In SS and inorganic trace mineral supplemented group, chicks fed 0.08 mg Se/kg had the lowest T3 concentration (*P* < 0.05) compared with not supplemented group, but did not differ from birds fed 0.16, 0.24, and 0.32 mg Se/kg diet. Differences between mineral sources in the T3 concentration were observed only when no Se was added to the diets (*P* < 0.05), with the inorganic minerals having higher T3. A trend (*P* = 0.089) in the interactive effects of Se levels and sources occurred in the free T3 (fT3) concentration in the serum, with a higher value for the organic treatment at only 0.16 mg Se/kg. The fT3 concentration in broiler chicks fed SY and organic trace microminerals tended to respond quadratically to increased Se supplementation (*P* = 0.091) with maximum value at 0.16 mg Se/kg diet. A quadratic (*P* < 0.05) or trend to quadratic (*P* = 0.057) change in free T4, was observed in the blood of birds fed SS and SY, respectively, with the highest values at 0.16 mg Se/kg diet. A linear increase (*P* = 0.037) in the free T3 to free T4 ratio occurred with SY supplementation.

**Table 2:** Effects of dietary selenium levels and sources on blood parameters of 17-d-old broiler chickens

Selenium source^1^	Selenium levels, mg/kg					Means	SEM^2^	*P-value*				
	0.0	0.08	0.16	0.24	0.32			Source	Level	S X L^3^	L^4^	Q^5^
***Glutathione peroxidase, U/L***												
Means	79.1 D	153 C	216 BC	266 AB	319 A		24.3	<0.01	<0.01	0.121	<0.01	0.406
SS	93.8 C	165 BC	240 AB	335 Aa	334 A	233					<0.01^e^	0.174
SY	62.8 C	126 BC	194 B	198 Bb	304 A	183					<0.01^e^	0.904
***Superoxide dismutase, U/L***												
Means	98.7	88.9	92.1	81.9	92.9		7.17	0.524	0.647	0.394	0.647	0.243
SS	98.5	86.4	91.8	88.0	93.4	91.6					0.887	0.187
SY	98.9	91.5	92.5	75.8	92.3	90.2					0.420	0.549
***Total T3, ng/ml***												
Means	3.32	2.30	3.01	2.51	2.73		0.125	0.419	0.056	0.134	0.278	0.241
SS	3.99 Aa	2.41 B	3.39 AB	2.59 AB	2.84 AB	2.87					0.136	0.018
SY	2.76 b	2.16	2.75	2.45	2.59	2.68					0.816	0.682
***Free T3, ng/ml***												
Means	0.0056	0.0045	0.0059	0.0059	0.0054		0.0002	0.336	0.270	0.089	0.344	0.786
SS	0.0060	0.0042	0.0050 b	0.0060	0.0055	0.0053					0.648	0.163
SY	0.0052 AB	0.0048 B	0.0068 Aa	0.0059 AB	0.0053 AB	0.0056					0.343	0.091
***Free T4, ng/ml***												
Means	31.6	36.4	38.8	32.9	35.9		0.942	0.416	0.050	0.403	0.627	0.140
SS	34.0	37.5	42.7	34.5	37.5	35.2					0.604	0.048^e^
SY	29.3	36.0	37.2	31.0	34.3	36.6					0.805	0.057^e^
***Free T3:Free T4***^***6***^												
Means	1.58	1.35	1.44	1.87	1.72		0.226	0.230	0.078	0.735	0.070	0.125
SS	1.60	1.36	1.28	1.79	1.53	1.51					0.575	0.531
SY	1.56	1.34	1.59	1.95	1.91	1.67					0.037	0.451

^1^ *SS* sodium selenite, *SY* selenium enriched-yeast

^2^ Standard error of means

^3^ Interactive effects between selenium and trace minerals sources and selenium levels

^4^ Linear effect of supplementation selenium levels

^5^ Quadratic effect of supplementation selenium levels

^6^ Free T3:Free T4 x 10000

^A-B^ Different uppercase letters in the same line and lowercase letters in the same column are different by Tukey test at 5%

^e^ Different superscripts letter indicates difference in the orthogonal contrasts between equations

### Se balance

A linear response (*P* < 0.01) was observed for the main effects of Se Intake (SeI), Se Carcass (SeC), Total Se (SeT) and Se Retention (SeR) (Table 3) as supplemental Se levels changed, regardless of Se source. A linear increase (*P* < 0.01) and quadratic response (*P* < 0.01) with steeper response at low Se intakes was observed for SeF with increasing Se supplementation levels. A quadratic increase (*P* < 0.01) was verified for SeF with SY supplementation and organic trace minerals, and, according to the fitted polynomial quadratic model SeF_(SY)_ = − 0.2641 × ^2^ + 0.1824x + 0.0245, *r*^2^ = 0.26, the maximum Se retention in the feathers was estimated at the dietary supplemental Se concentration of 0.30 mg Se/kg. The lowest supplemental levels of Se in feeds that promoted maximum SeF were 0.16 and 0.08 mg Se/kg for birds fed SS (*P* < 0.05) and SY (*P* < 0.05), respectively. When a comparison is made between not supplemented group and SY supplemented chicks, greater SeR in was observed at 0.16 and 0.32 mg Se/kg in birds fed SS (*P* < 0.05), meanwhile in the SY fed chicks, SeR was higher at 0.24 and 0.32 mg Se/kg (*P* < 0.05). A trend (*P* = 0.075) for a linearly decreased response was observed in the SeBal with the increasing Se levels.

**Table 3:** Effects of dietary selenium levels and sources on selenium balance on 17-d-old broiler chickens

Selenium source^1^	Selenium levels, mg/kg					Means	SEM^2^	*P-value*				
	0.0	0.08	0.16	0.24	0.32			Source	Level	S X L^3^	L^4^	Q^5^
***Intake, mg/bird***												
Means	0.065 E	0.099 D	0.139 C	0.176 B	0.210 A		0.003	0.794	<0.01	0.794	<0.01	0.882
SS	0.067 E	0.098 D	0.138 C	0.175 B	0.211 A	0.138					<0.01^e^	0.435
SY	0.064 E	0.099 D	0.141 C	0.176 B	0.209 A	0.138					<0.01^e^	0.321
***Carcass, mg/bird***												
Means	0.050 C	0.062 BC	0.073 AB	0.075 AB	0.082 A		0.006	0.118	<0.01	0.717	<0.01	0.221
SS	0.046 B	0.062 AB	0.073 A	0.067 AB	0.078 A	0.065					<0.01^e^	0.261
SY	0.054 B	0.062 AB	0.074 AB	0.083 A	0.085 A	0.072					<0.01^e^	0.539
***Feathers, mg/bird***												
Means	0.0015 C	0.0020 BC	0.0023 AB	0.0025 A	0.0025 A		0.002	0.049	<0.01	0.725	<0.01	<0.01
SS	0.0015 C	0.0018 BC	0.0022 AB	0.0024 AB	0.0025 A	0.0021					<0.01^e^	0.331
SY	0.0015 B	0.0021 AB	0.0025 A	0.0027 A	0.0025 A	0.0023					<0.01^e^	<0.01
***Total, mg/bird***^***6***^												
Means	0.051 C	0.064 BC	0.077 AB	0.077 AB	0.084 A		0.006	0.136	<0.01	0.698	<0.01	0.168
SS	0.047 B	0.064 AB	0.077 A	0.069 AB	0.081 A	0.068					<0.01^e^	0.208
SY	0.055 B	0.064 B	0.077 A	0.085 A	0.088 A	0.074					<0.01^e^	0.488
***Retention, mg/bird***^***7***^												
Means	0.024 C	0.037 BC	0.050 AB	0.050 AB	0.057 A		0.006	0.136	<0.01	0.698	<0.01	0.168
SS	0.020 B	0.037 AB	0.050 A	0.042 AB	0.054 A	0.041					<0.01^e^	0.208
SY	0.028 B	0.037 AB	0.050 AB	0.058 A	0.061 A	0.047					<0.01^e^	0.488
***Balance, %***												
Means	34.1	34.0	35.3	25.6	27.4		4.55	0.393	0.393	0.487	0.075	0.421
SS	30.3	37.0	35.8	24.0	25.8	30.6					0.133	0.252
SY	37.9	31.1	34.7	33.2	29.0	33.2					0.301	0.997

^1^ *SS* sodium selenite, *SY* selenium enriched-yeast

^2^ Standard error of means

^3^ Interactive effects between selenium and trace minerals sources and selenium levels

^4^ Linear effect of supplementation selenium levels

^5^ Quadratic effect of supplementation selenium levels

^6^ Sum of the Se content in the feathers and carcass

^7^ Whole body Se in the birds in the beginning of the experiment: 0.0027 mg Se/kg

^A-B^ Different uppercase letters in the same line are different by Tukey test at 5%

^e^ Different superscripts letter indicates difference in the orthogonal contrasts between equations

### Se concentration in tissue

Se concentration in the liver showed a trend (*P* = 0.073) to a linear decrease with increased Se supplementation levels when considering both Se sources combined (Table 4). A linear reduction in the Se concentration in the liver was observed (*P* < 0.05) with increasing SY supplemented levels and organic trace minerals. Neither the Se sources nor levels influenced the Se concentration in the broiler breast muscle (*P* > 0.05).

**Table 4:** Effects of dietary selenium levels and sources on selenium tissue concentration of 17-d-old broiler chickens*

Selenium source^1^	Selenium levels, mg/kg					Means	SEM^2^	*P-value*				
	0.0	0.08	0.16	0.24	0.32			Source	Level	S X L^3^	L^4^	Q^5^
***Liver, mg/kg***												
Means	1.55	1.47	1.41	1.37	1.33		0.06	0.288	0.504	0.185	0.074	0.764
SS	1.41	1.50	1.50	1.54	1.44	1.47					0.790	0.600
SY	1.69 A	1.45 AB	1.36 AB	1.18 B	1.24 AB	1.38					0.006	0.343
***Breast muscle, mg/kg***												
Means	0.296	0.279	0.278	0.292	0.267		0.08	0.311	0.516	0.501	0.293	0.999
SS	0.307	0.258	0.272	0.292	0.253	0.277					0.214	0.700
SY	0.285	0.299	0.284	0.293	0.281	0.288					0.811	0.700

^*^ Values represented on a dry matter basis

^1^ *SS* sodium selenite, *SY* selenium enriched-yeast

^2^ Standard error of means

^3^ Interactive effects between selenium and trace minerals sources and selenium levels

^4^ Linear effect of supplementation selenium levels

^5^ Quadratic effect of supplementation selenium levels

^A-B^ Different uppercase letters in the same line are different by Tukey test at 5%

### Relative bioavailability estimates

**Fig. 1:**
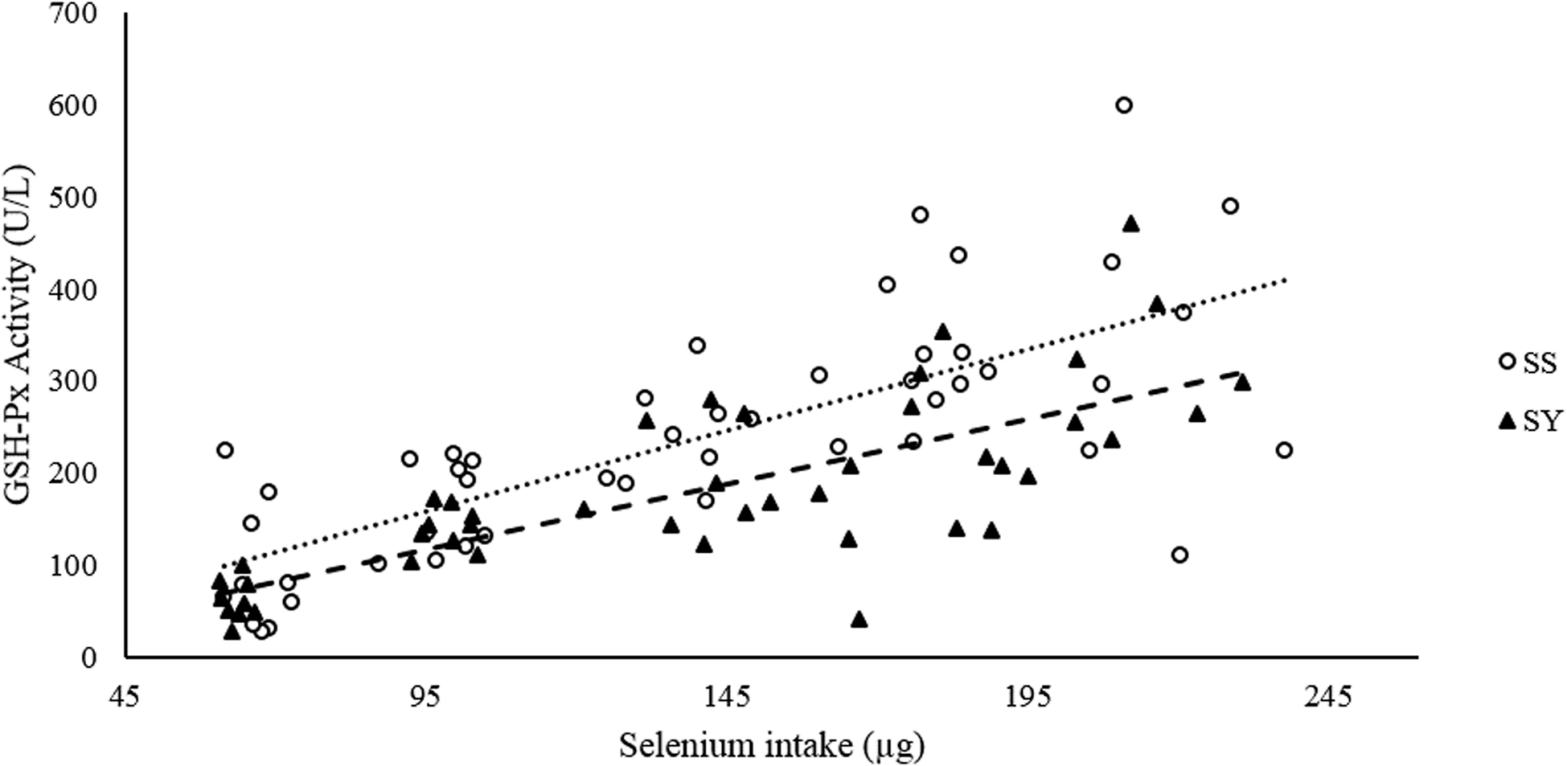
Relative bioavailability of SS and SY in the GSH-Px activity of 17 d old chicks. Inorganic (**•••**) and organic (**−−**) linear regressions. GSH-Px_(SS)_ = 1756.8x – 8.6073; *R*^2^: 0.54. GSH-Px_(SY)_ = 1415.2x – 17.477; *R*^2^: 0.58. SS relative bioavailability: 100%. SY relative bioavailability: 81%

**Fig. 2:**
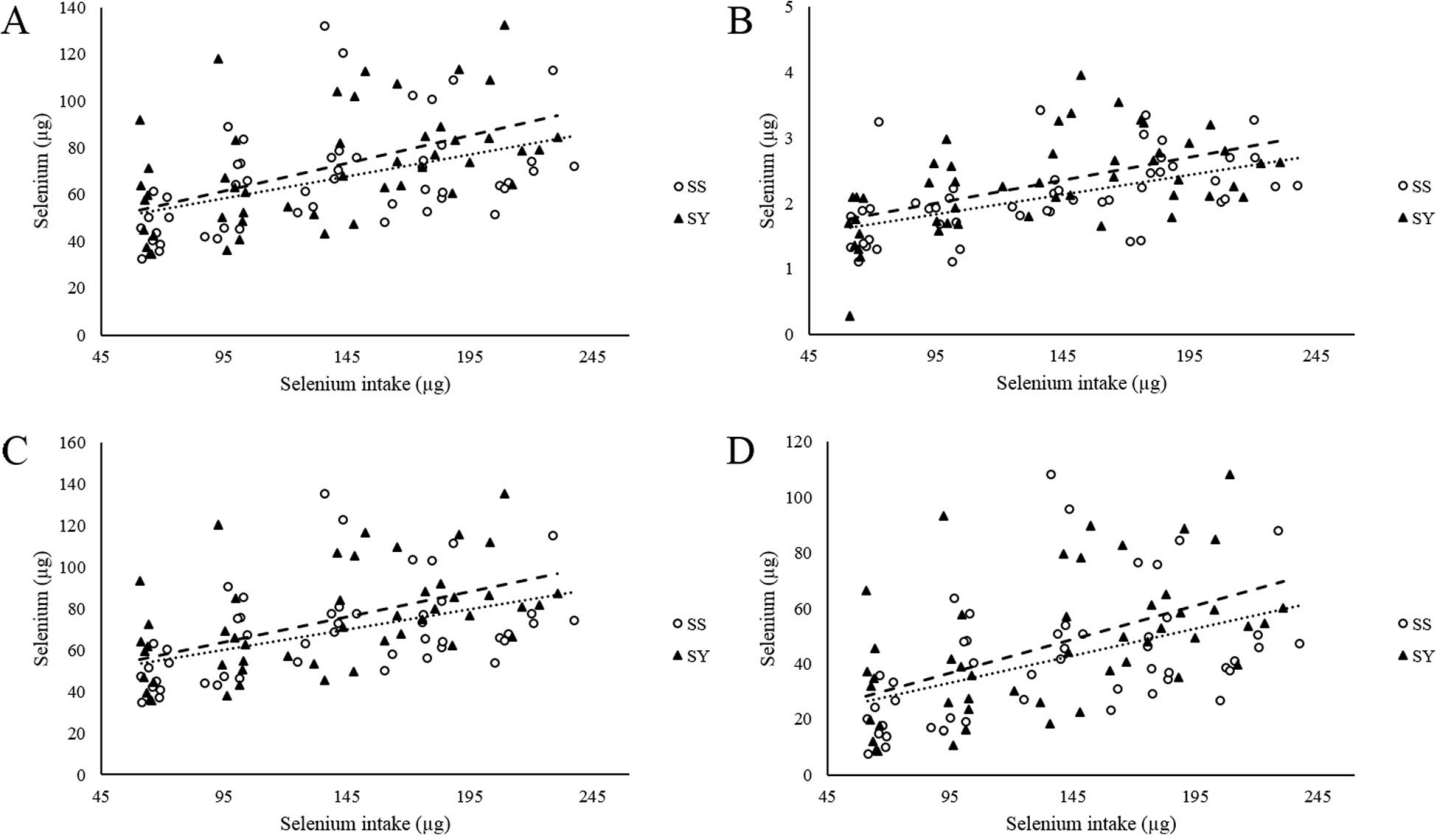
Relative bioavailability of SS and SY in the Se retention of 17 d old chicks. Inorganic (**•••**) and organic (**−−**) linear regressions of Se retained in the carcass (**a**), feathers (**b**), total (**c**) and retention (SeTf-SeTi) (**d**). SeC_(SS)_ = 0.1879x + 0.041; *R*^2^: 0.20. SeC_(SY)_ = 0.2368x + 0.039; *R*^2^:0.27. SeF_(SS)_ = 0.006x + 0.0013; *R*^2^: 0.30. SeF_(SY)_ = 0.007x + 0.0013; *R*^2^: 0.29. SeT_(SS)_ = 0.1939x + 0.0419; *R*^2^: 0.21. SeT_(SY)_ = 0.2439x + 0.0404; *R*^2^: 0.28. SeR_(SS)_ = 0.1939x + 0.0148; *R*^2^: 0.21. SeR_(SY)_ = 0.2439x + 0.0133; *R*^2^: 0.28

Multiple linear regression equations were not utilized to evaluate the relative bioavailability in this experiment because the basal diets were different, with different mineral sources, so it would be invalid to force a common intercept. Significant (*P* < 0.05) linear regression equations in the same variable for the two Se sources were observed for the GSH-Px activity, SeC, SeF, SeT as well as SeR (Figs. 1 and 2). When the response to SS was set to 100%, the estimated relative bioavailability of SY were 81, 126, 117, 126 and 126% based on the retention variables SeC, SeF, SeT and SeR, respectively, indicating greater SY availability to improve these corresponding parameters.

## Discussion

This experiment was designed to assess the effects of supplemental Se levels, trace minerals sources, and their interaction on the performance, Se retention, antioxidant enzymatic activity, and serum concentration of thyroid hormones of growing broilers chicks.

Selenium has been recognized as a trace element of physiological importance for growing chicks, given its participation in pathways, which include antioxidant responses and hormone secretion [1]. Nonetheless, despite such essentiality, our outcomes revealed that neither supplemental Se level nor Se source (SS and SY) seemed to have a remarkable effect on growing chick performance. Such outcomes support previous research findings that Se supplementation, regardless of the source (inorganic or organic), does not affect broiler performance traits [3, 17–19], but differ from those reported by Choct et al. [2], Cemin et al. [22], and Markovic et al. [27] who observed positive effects of Se supplementation in growing chick BW and/or FCR. Establishing optimal in-feed Se supplementation for growing broilers has been revealed to be particularly challenging, since estimates may be affected by several factors, such as Se source, the concentration and source of the other trace minerals in experimental diets, as well as Se concentration in feed ingredients, which may range to great extent due to Se content in soil [22] In the current research, even though the amount of Se provided by the basal diets was lower than the requirement estimate from NRC [21] for growing chicks (0.123 and 0.131 vs. 0.150 mg Se/kg), such concentrations were sufficient to allow growth rates similar to those in chicks fed the highest Se levels under study. Presumably, the reasons underlying the absence of chick growth response to Se supplementation in the current research may be associated with the redox status of the birds throughout the feeding assay and with residual effects of maternal nutrition on chick storage of Se. Selenium is mainly required as a cofactor for enzymes involved in ROS scavenging, and therefore, the higher the exposure of birds to stressors, the higher the Se requirement for metabolism is expected to be. The stressors commonly highlighted as those of significant impact on poultry redox balance have been inadequate temperature and ventilation, excessive bird handling, high stocking density, sanitary challenges and imbalanced trace mineral nutrition [1]. In the current research, chicks were housed in an environmentally controlled facility and managed according to the genetic strain guideline. Moreover, experimental diets were formulated to meet chick requirements, except for Se, and no clinical signs of sanitary challenge were noticed throughout the assay. Presumably, such experimental conditions avoid the disruption of redox balance in birds, and consequently, minimized the utilization of Se in antioxidant pathways. Another point worth of note, one whose relationship with oxidative stress may influence the Se utilization and the physiological demands for this element, is the age of chicks used in our assay. Because young chicks are not so susceptible to oxidative stress compared with broilers at slaughter age, the metabolic needs for Se are expected to be lower in the starter phase. Finally, Se status of chicks at hatch, influenced by breeder nutrition, as well as Se content of pre-experimental diets may have provided enough Se for physiological needs, so the 7-d-experimental period may have been too short to detect nutritional signs of Se deficiency in chicks fed Se-deprived diets. As an attempt of estimating the optimal supplemental Se level for chicks based on growth performance responses, BW and ADG data were fitted to a polynomial regression model, being optimized at 0.133 and 0.130 mg Se/kg diet, respectively, in chicks fed Se-Yeast. These estimates are lower than the value described by NRC [21] of 0.15 mg/kg but similar to the values recommended by Rostagno et al. [28] of 0.138 mg Se/kg for organic Se.

As a cofactor for 5′-deiodinase (5′-ID), Se participates in the conversion of T4 to the biologically active T3, affecting the metabolism of protein, lipids and carbohydrates [7, 9, 10, 29]. The influence of Se on thyroid functioning was well illustrated by Jianhua et al. [6] who noticed a decrease in the synthesis of T3 in chicks fed diets supplemented with iopanoic acid, a monodeiodinase inhibitor, which depresses the hepatic 5′-ID activity. Yet, the authors noticed a negative correlation of T3 plasmatic concentration with chick growth performance. In the current study, a positive relationship was noticed between the thyroid hormones and growth performance. As detailed in Table 2, serum concentration of free T3 of chicks fed SY and organic trace mineral premix exhibited a similar pattern to those noticed for ADG (Table 1). The linear increase in the free T3: free T4 ratio in chicks fed SY supplemented diets indicates an increase in the conversion of free T3 from the inactive free T4. Such findings support those reported by Madkour et al. [9] and Wang et al. [10] that noticed higher T3 and low T4 serum concentrations in response to Se supplementation levels and organic Se sources (selenium yeast and dl-selenomethionine). The influence of selenium yeast supplementation on the thyroid hormone concentrations agrees with the findings of Upton et al. [8] who reported a possibly more efficient conversion of T4 into T3 after selenium yeast supplementation.

Glutathione peroxidase, as well as superoxide dismutase and catalase, is a component of the antioxidant system responsible for neutralizing ROS species, which may potentially damage cells and tissues [2, 5]. Although organic Se has been considered more bioavailable than inorganic sources when considering GSH-Px activity [5, 26], we noticed that chicks fed diets containing selenium yeast and organic trace minerals exhibited lower values of GSH-Px activity in the blood compared with SS fed chicks (Fig. 1). Our outcomes support previous findings that chicks fed SS supplemented diets exhibited a higher GSH-Px activity in the plasma, kidneys, pancreas and breast muscles compared with birds fed diets containing selenium yeast [2, 3, 17, 25, 30–32]. Even though after being absorbed in the intestine, either inorganic or organic Se may be used in GSH-Px activation, the sources differ regarding the metabolic pathways necessary to be incorporated into selenoproteins [33, 34]. After absorption, Se from SS is converted in the liver into hydrogen selenide (H_2_Se), a compound that plays a central role in Se metabolism. Once formed, H_2_Se may be either methylated and excreted or converted to selenophosphate (HSePO_3_^2−^), whose Se will be incorporated into tRNA Se-cysteinyl with further translation to selenoproteins, such as GSH-Px [35]. On the other hand, Se-Met present in selenium yeast follows a transsulfuration into Se-Cys through a B_6_-dependent reaction [36]. Unlike, Se-Met, Se-Cys, a component of GSH-Px enzyme family as well as other selenoproteins (e.g. deiodinases, thioredoxin reductases, selenophosphate synthetase, etc.) cannot be directly incorporated into proteins without being previously converted into H_2_Se. Thus, the conversion of Se-Met into H_2_Se requires more steps than selenite, which may explain the lower efficiency of organic Se utilization to produce such selenoproteins, i.e. Se-Cys containing enzymes and proteins.

As detailed in Table 3, the concentration of Se in the feathers of chicks fed selenium yeast were higher than birds fed SS supplemented diets, which suggests that Se provided by the organic source under study, i.e. selenium yeast, were preferably utilized in feather protein synthesis, instead of being used for GSH-Px synthesis. Our findings demonstrated that selenium yeast supplementation increased the content of Se in chick feathers by 9.5% compared to SS group. These outcomes support those reported by Mahan and Parret [35], Edens et al. [37], Choct et al. [2], Yoon et al. [31] and Couloigner et al. [19]. Similarly, Edens et al. [37] and Choct et al. [2] reported a higher Se retention in selenium yeast fed chicks. Yet, the referred authors correlated such fact with improvements noticed in feathering score, which was addressed to the utilization of Se-Met for the synthesis of keratin, the major structural protein in feathers, skin, beak, and claws [38]. In eukaryote cells, both methionine (Met) and Se-Met are recognized and utilized in the same way since there is not a specific tRNASe-Met in cells, which is equivalent to say that the incorporation of Se-Met into bodily proteins is a non-specific event in which Met is replaced by Se-Met happens in tRNAMet during protein synthesis [39].

Although the content of Se in carcasses and feathers, as well the Se retention, were linearly increased as supplemental Se levels increased (Table 3), the sources of Se under study affected only the Se content in feathers. As detailed in Table 3, Se intake was linearly increased as supplemental Se levels increased, which is expected since ADFI was not affected by Se supplementation. We noticed, however, that compared to Se intake responses, Se carcass and feather deposition increased at a lower extent as Se supplementation increased, reaching a plateau phase at 0.160 mg Se/mg diet, which was also observed for GSH-Px and thyroid hormone responses. The Se concentration in the tissues is level and source dependent [19, 26, 30]. In the present study, chicks fed diets containing selenium yeast and organic trace minerals exhibited a linear reduction in the liver Se concentration. Liver is the primary Se storage organ and its Se concentration has been used as a criterion to assess the Se status in the body [32]. Dietary Se-Met is not entirely converted into selenoproteins and may be stored in organs with high protein synthesis such as the skeletal muscle, liver and kidney [40], whereas SS cannot be stored in the body [4]. Our data (Table 3) indicate that not only did the Se retained in the broiler chick body increased with the increased Se levels, but also the Se retained in the feathers was greater when birds were fed selenium yeast and organic trace minerals, which suggests that the reduction in the Se concentration in the liver was due to its deposition as methionine in other tissues. Such evidences confirm that SY molecule was preferably utilized as a source of Met instead of Se. In the liver, Met could have different metabolic fates, which include reaching blood stream to be utilized in body and feather protein accretion, remaining in the liver to be used in methyl donor reactions in Met cycle, or finally, being split into carbon skeleton and ammonia, which would be further excreted as uric acid [41]. Although Se is essential for proper liver function, high hepatic concentration of Se may adversely affect hepatocytes. Hao et al. [42] noticed an increased hydrogen peroxide production when supplementing broiler diets with 0.62 mg nano-Se/kg, which, in turn, as highlighted by the authors, reflected greater serum concentrations of aspartate transaminase (AST) and alanine transaminase (ALT), enzymes that are biomarkers of hepatic lesions [43, 44]. In turn, Peric et al. [23] observed that when supplied at high levels, SS increased serum concentrations of AST and ALT in broilers compared with selenium yeast fed birds. The enrichment of meat products with Se is desired due to its benefits in the maintenance of redox balance of cells and tissues, which may improve that final quality of meat products. Unexpectedly, our outcomes indicate that neither Se source nor supplemental Se levels influenced Se deposition in chick breast muscle. Conversely, Wang and Xu [25] and Sevcikova et al. [45] reported an increase in the Se concentration in broiler breast muscle in response to Se supplementation. As highlighted by the referred authors, Briens et al. [26], Sevcikova et al. [45], discrepancies found in literature regarding tissue Se concentration might be explained by the experimental period length, calculation method and analytical method to determine the Se concentration.

The bioavailability of selenium yeast compared with SS is described in Fig. 2. According to the slope ratio between sources, the relative bioavailability of selenium yeast compared with SS for the Se content in carcass, feathers, total and Se retention were, 126, 116, 125 and 125%, respectively. Ammerman et al. [46] defined the term “bioavailability” as the degree to which an ingested nutrient is absorbed and utilized in the metabolism by the animal, so the higher the bioavailability of a given nutrient source, the lower its amount to be supplemented in diets will be. Based on our findings, more SS needs to be included in diets to reproduce similar retention in chick tissues, which is undesired on environmental grounds due to higher excretion rates. Additionally, excessive SS has shown to have pro-oxidant properties and lead to hepatic [42] and intestinal damage [47]. Despite its greater bioavailability, our evidence demonstrates that organic Se in the feathers indicates that the Se-Met from selenium yeast was utilized as a source of Met and/or Cys rather than Se itself. Because our evidence indicates that organic Se, i.e. selenium yeast, was used as Met for feather proteins, playing therefore a structural role, selenium yeast supplemented diets could have slightly exceeded chick needs for Met.

## Conclusions

To the best of our knowledge, this is the first research conducted to investigate supplemental Se levels and trace mineral sources on growing chick responses, where the other trace minerals had the same nature of the Se source assessed, i.e. organic or inorganic. Irrespective of the source, based on performance traits, the supplemental Se level for growing chicks under the conditions of this study was lower than the requirement estimate of NRC [21]. The ideal level of selenium yeast, provided as SY in an organic trace mineral mix, for growth was 0.133 mg Se/kg diet, whereas for SS, the benefits of its supplementation on growth were not so clear. Selenium, regardless of the source, proved to be important in antioxidant responses and thyroid hormone activation. Selenium utilization by chicks differed between the sources assessed, and outcomes suggested that selenium yeast is less efficiently used in antioxidant pathways compared with SS, but more efficiently retained into bodily proteins. Based on Se storage, SY, provided as Sel-Plex®, showed a higher relative bioavailability compared to SS.

## Methods

### The current study was carried out in compliance with the ARRIVE guidelines

The procedures involving animal care and use were previously approved by Ethics on Animal Use Committee of the Federal University of Viçosa, Viçosa, Minas Gerais, Brazil, prior to the beginning of the assay (Register number 111/2014).

### Birds and husbandry

A total of five hundred 1-d-old male Cobb 500 chickens were obtained from a local commercial hatchery (Rivelli, Mateus Leme, Brazil) and used in the current assay. From 1 to 7 d of age, birds were fed a pre-starter diet formulated to meet or exceed Rostagno et al. [28] nutritional recommendations, except for Se, whose dietary supplementation was provided according to the NRC [21]. Throughout the entire pre-experimental period, all chicks had free access to water and feed (mash). At 8 d of age, chicks were housed in an environmentally controlled room and allotted in a complete randomized design into 0.49 m × 0.27 m × 0.33 m (length x height x width) plastic cages with raised wire floors until the end of the feeding assay. Initial stocking density corresponded to 30.9 chicks/m^2^). Feed and demineralized water were provided ad libitum throughout the 10-day experimental period. Photoperiod was set at 12 h natural light/12 h artificial light. Prior to the experimental period at day 8 of age, all chicks were weighed and assigned to treatment groups so initial body weight (180.3 ± 1.18 g) was similar among experimental treatments.

### Experimental diets and treatments

A 2 × 5 factorial arrangement was used to investigate the effect of 2 sources of microminerals and Se (as organic and inorganic sources) and 5 Se supplementation levels (0, 0.08, 0.16, 0.24, and 0.32 mg Se/kg). Ten replicate cages of 5 chicks were randomly assigned to each of 10 treatment groups. Each cage was considered an experimental unit. Selenium sources included supplemental Se from selenium selenite (SS; 45.85% Se - Metalloys & Chemicals commercial Ltda - Cotia, SP) and selenium enriched yeast (SY; 0.2405% Se – Sel-Plex® - Alltech, Maringá, Brazil). Organic trace microminerals were supplied as Bioplex®Fe (15% Fe), Bioplex®Zn (15.05% Zn), Bioplex®Cu (11.36% Cu), Bioplex®Mn (13.86% Mn), whereas inorganic trace minerals sources included iron sulphate (21.91% Fe), zinc sulphate (22.95% Zn), copper sulphate (25.13% Cu), and manganese sulphate (30.3% Mn), and I was added as calcium iodide (86% I) in both trace mineral supplements. Therefore, diets containing SS and SY were supplemented with inorganic and organic trace mineral supplements, respectively. A semi-purified basal diet, based on casein, albumin, corn, and dextrose (Table 5), was formulated to meet or exceed nutritional requirements of Rostagno et al. [28] for starter broilers (8–21 d of age), except for trace minerals. From the basal diet, 4 different diets were produced.

**Table 5:** Nutritional composition of semi purified diets used in experimental period, as-fed basis

Ingredients	g/kg
Corn	300
Albumin^a^	120
Starch	127.4
Dextrose	134
Casein^a^	40
Soy protein isolate	40
Broken rice	80
Soybean oil	20
Cellulose^a^	40
Calcium carbonate^a^	17.6
Potassium phosphate^a^	15.80
Magnesium chloride^a^	6.50
Potassium chloride^a^	4.68
Choline chloride, 60%	3.75
Mixture of amino acids^b^	35.55
Micronutrients^c^	12.65
Microminerals^d^	2.00
Phytase^e^	0.10
*Calculated nutrients*	
AMEn, kcal/kg	3122
Crude protein^f^, %	22.58
*SID amino acids, %*	
Lysine, %	1,254
Methionine, %	0.552
Methionine + Cystine, %	0.913
Threonine, %	0.831
Calcium^f^, %	0.878
Total P^f^, %	0.581
Available P, %	0.420
Se^f^, mg/kg	0.138

^a^ P.A purism reagent exceeds standard ACS specification in trace metals analysis

^b^ 0.03% L-lysine (79%); 0.27% L-arginine (98.5%); 0.40% L-glycine (98.5%); 0.85% L-alanine (99%); and 2.0% L-glutamic acid (99%). The amino acids alanine, glycine, and glutamic acid were added to maintain the ratio of essential nitrogen to total nitrogen at 0.50

^c^ 0.055% coccidiostatic; 0.010% avilamycin; 0.030% BHT; 1.02% sodium phytate and 0.150% vitamin blend supplemented per kg of feed: vitamin A, 7500 IU; vitamin D3, 1900 IU; vitamin E, 28 IU; vitamin B1,2 mg; vitamin B2, 5 mg; vitamin B6, 1.2 mg; vitamin B12, 12 mcg; vitamin K, 1.5 mg; nicotinic acid, 0.03 mg; pantothenic acid, 0.01 mg; folic acid, 0.7 mg; and biotin, 0.07mg

^d^ Trace mineral supplemented per kg of feed: 80 mg Fe; 1 mg I; 60 mg Mn; 40 mg Zn; and 10 mg Cu, except for Se added according each experimental treatment as 0; 0.08; 0.16; 0.24 and 0.32 mg Se. Inorganic trace mineral supplement sources: Ferrous Sulphate (21.91% Fe), Zinc Sulphate (22.95% Zn), Copper Sulphate (25.13% Cu), Calcium Iodide (86% I), Manganese Sulphate (30.3% Mn) and Sodium Selenite (51,6% Se). Organic trace mineral supplement sources: Bioplex®Fe (15% Fe), Bioplex®Zn (15.05% Zn), Bioplex®Cu (11.36% Cu), Calcium Iodide (86% I), Bioplex® Mn (13.86% Mn), Selplex® selenium enriched-yeast (0.236% Se)

^e^ Microbial phytase – 600 FTU/kg

^f^ Analyzed value in ingredients

The 4 diets differed from each other with regard to amount and source of Se supplemented (SS or SY) and to the trace mineral supplemented (organic trace mineral or inorganic trace mineral) as follows: 1) basal diet supplemented with organic trace mineral (BD-OTM) without Se supplementation; 2) basal diet supplemented with organic trace mineral + 0.32 mg Se/kg feed supplemented as SY; 3) basal diet supplemented with inorganic trace mineral without Se supplementation; and 4) basal diet supplemented with inorganic trace mineral + 0.32 mg Se/kg of feed supplemented as SS. Diets supplemented with organic trace mineral supplement containing supplemental Se at 0 and 0.32 mg/kg as SY were mixed to produce five dilution series, which, in turn, resulted in five different organic Se concentrations in diets with organic trace minerals: 0, 0.08, 0.16, 0.24, and 0.32 mg Se/kg of feed. The same procedure was adopted with diets supplemented with inorganic trace mineral, resulting in the five levels of inorganic Se with inorganic trace minerals and a total of 10 diets. Except for Se, all microminerals were supplied to meet NRC [21] required estimates. Sodium phytate as well as a commercial phytase enzyme were added to the semi-purified diets to simulate practical cereal-based diets. Diets were analyzed for Se content prior to the beginning of the assay through the method described below. In order to ensure the reliability to our data, the commonly accepted as the minimum number of samples were collected as described below.

### Performance measurements and tissue mineralization

The birds were weighed, and the experimental diets were introduced when the birds were 8 days of age. At 17 d of age, all chicks and feed leftovers from each experimental unit were weighed to determine body weight (BW) and average daily feed intake (ADFI). Average daily gain (ADG) and feed conversion ratio (FCR) were calculated from such data. Mortality rate was also monitored. At the end of the assay, 1 chick per cage (10 birds/treatment) was randomly selected and slaughtered by cervical dislocation. The bird had the liver and left side of breast muscle collected, lyophilized for 72 h at − 80 °C under 800 mbar of pressure (Liobras– São Carlos, SP), ground in a ball mill (Tecnal Equipamentos para Laboratório, TE-350, São Paulo, Brazil) and stored for further analysis of Se content [48].

### Selenium retention

At the beginning of the trial, a reference group of ten 8-d-old chicks were randomly selected and slaughtered after a 12 h fasting period (water was provided ad libitum). Similarly, one chick from each experimental unit was randomly selected and slaughtered at the end of the experimental period (17 d of age). Birds were weighed before and after plucking to determine the weight of feathers. The carcasses were frozen in liquid nitrogen and ground in an industrial mixer (Spolu – Benesse do Brasil - Itajobi, SP) and stored for further analysis of Se content. The whole-body Se retention (SeR) and balance (SeBal) were calculated as follows:

$$ \mathrm{SeR}=\mathrm{SeTf}-\mathrm{SeTi}, $$

$$ \mathrm{SeBal}\left(\%\right)=\mathrm{SeR}/\mathrm{SeI}\times 100, $$

where SeTf and SeTi are the Se amount (mg) in the carcass (SeC) and feathers (SeF) at the end and beginning of the experimental period, respectively, and SeI is the Se intake (mg) during the same period.

### Selenium concentration in the tissues

The Se concentration of the feed ingredients, experimental diets and tissues were analyzed at the Mineral Laboratory of the Animal Science Department of the Universidade de São Paulo (São Paulo, SP) following the methodology proposed by Olson et al. [48]. The Se levels of all the ingredients were determined before diet provision to safely estimate the minimum mineral values.

### Antioxidant enzymes and hormones concentration

At the end of the experimental period one animal per cage was randomly selected to collect blood samples through heart puncture. The samples were collect in three tubes, heparinized vacutainer tubes containing Na heparin to analyze the whole blood activity of GSH-Px, vacutainer tubes containing EDTA to analyze the activity of superoxide dismutase (SOD), and vacutainer serum tubes for the thyroid hormones (T3 and T4) analysis, respectively. The enzymatic activity was determined through the kits of Randox Laboratories Ltda. (County Antrim, UK) Ransel® and Ransod®, respectively, following the manufacturer guidelines. Total thyroxine and free and total triiodothyronine in the serum were analyzed at Diagnóstico do Brasil (São José do Rio Preto, SP) laboratory through the immunoassay kits of Beckman Coulter Diagnostics, Access Total T4®, Access Free T3® and Access Total T3®, respectively.

### Statistical analysis

Data were analyzed as a completely randomized design under an incomplete 2-way (source x levels) factorial assay with inorganic and organic trace minerals supplement without selenium supplementation and Se supplement levels as SY in organic trace minerals supplement and SS in inorganic trace minerals supplement. In this context, given the implied issues of level zero from both sources, the traditional two-way factorial analysis was generalized to a fractional factorial design, which consists in a carefully chosen subset (fraction) under an experimental treatments framework [49]. This approach is easily accomplished by using common statements from PROC MIXED of SAS® (SAS Institute Inc., Cary, NC) software. According to the previously mentioned analysis, the significance (*P* < 0.05) of source effect (only two levels) was evaluated through F-test; whereas orthogonal contrasts were applied to perform the analysis between linear and quadratic responses of dependent variables in function of increasing Se levels. Also, the effects of Se levels were compared using Tukey’s multiple comparison test. The cages average served as the experimental unit for growth performance, while the single chicks (one per cage) served as the experimental unit for tissue mineral contents, retention, thyroid hormone concentration and enzyme activities. Relative bioavailability values of Se as selenium yeast were estimated by slope ratio comparison based on independent linear regressions using SS as the standard source. The regressions were calculated using the supplemental Se intake (adjusted by feed intake during the whole experiment) as the independent variable rather than added Se level. A tendency was considered for *P*-value between 0.05 and 0.1.

## Data Availability

The dataset generated and/or analyzed during the current study is not publicly available since the data is a preliminary part of another study. The data is, however, available from the corresponding author on reasonable request.

## Abbreviations

Se: Selenium
SY: Selenium-yeast
SS: Sodium selenite
BW: Body weight
ADG: Average daily gain
ADFI: Average daily feed intake
FC: Feed conversion
GSH-Px: Glutathione peroxidase
SOD: Superoxide dismutase
Se-Cys: Selenocysteine
Se-Met: Selenomethionine
ROS: Reactive oxygen species
T4: Thyroxin
fT4: Free T4
T3: Triiodothyronine
fT3: Free T3
NRC: National Research Council
SeI: Selenium intake
SeC: Selenium in the carcass
SeT: Selenium total
SeR: Selenium retention
SeF: Selenim in the feathers
SeBal: Selenium balance
5′-ID: 5′-deiodinase
H_2_Se: Hydrogen selenide
HSePO_3_^2−^: Selenophosphate
